# Rapamycin Attenuates Splenomegaly in both Intrahepatic and Prehepatic Portal Hypertensive Rats by Blocking mTOR Signaling Pathway

**DOI:** 10.1371/journal.pone.0141159

**Published:** 2016-01-06

**Authors:** Yunyang Chen, Weijie Wang, Huakai Wang, Yongjian Li, Minmin Shi, Hongwei Li, Jiqi Yan

**Affiliations:** 1 Department of Surgery, Ruijin Hospital, Shanghai Jiaotong University School of Medicine, Shanghai, China; 2 Shanghai Institute of Digestive Surgery, Ruijin Hospital, Shanghai Jiaotong University School of Medicine, Shanghai, China; 3 Department of Surgery, First Affiliated Hospital of Zhengzhou University, Zhengzhou University, Zhengzhou, China; Texas A&M Health Science Center, UNITED STATES

## Abstract

**Background:**

Spleen enlargement is often detected in patients with liver cirrhosis, but the precise pathogenetic mechanisms behind the phenomenon have not been clearly elucidated. We investigated the pathogenetic mechanisms of splenomegaly in both portal hypertensive patients and rats, and tried to identify the possible therapy for this disease.

**Methods:**

Spleen samples were collected from portal hypertensive patients after splenectomy. Rat models of portal hypertension were induced by common bile duct ligation and partial portal vein ligation. Spleen samples from patients and rats were used to study the characteristics of splenomegaly by histological, immunohistochemical, and western blot analyses. Rapamycin or vehicle was administered to rats to determine the contribution of mTOR signaling pathway in the development of splenomegaly.

**Results:**

We found that not only spleen congestion, but also increasing angiogenesis, fibrogenesis, inflammation and proliferation of splenic lymphoid tissue contributed to the development of splenomegaly in portal hypertensive patients and rats. Intriguingly, splenomegaly developed time-dependently in portal hypertensive rat that accompanied with progressive activation of mTOR signaling pathway. mTOR blockade by rapamycin profoundly ameliorated splenomegaly by limiting lymphocytes proliferation, angiogenesis, fibrogenesis and inflammation as well as decreasing portal pressure.

**Conclusions:**

This study provides compelling evidence indicating that mTOR signaling activation pathway plays a key role in the pathogenesis of splenomegaly in both portal hypertensive patients and rats. Therapeutic intervention targeting mTOR could be a promising strategy for patients with portal hypertension and splenomegaly.

## Introduction

Splenomegaly is a frequent finding in many kinds of chronic liver diseases as a consequence of portal hypertension (PHT) [1]. It usually manifests with a severe hypersplenism, characterized by a significant reduction in one or more of the cellular elements of the blood, which will lead to anemia, thrombocytopenia and even life threatening complications like esophageal variceal hemorrhage [1–3]. Episodes of splenic ischemia and infarction can also be detected in the enlarged spleen [1]. Even minor trauma can be a hazard to splenic rupture [1]. Importantly, splenomegaly is not only as a silent consequence but also a proactive contributor that congests the portal venous system and participates in the maintenance and aggravation of portal pressure, which may contribute to gastroesophageal varices and related bleeding [4, 5]. To date, however, limited effective medical therapies have been reported for splenomegaly and its relative complications. The precise pathogenetic mechanisms leading to spleen enlargement in PHT have been yet poorly understood [1, 3, 4].

In traditional concept, the enlargement of spleen in liver cirrhotic patients, also known as passive congestive splenomegaly, is due to the increased portal venous resistance that kidnaps the red blood cells pooling in the red pulp [4]. However, it has been challenged as conflicting data have been published in this field. As emerging data showed, besides the evident pooling of blood in the red pulp, a combination of angiogenesis and fibrogenesis, as well as hyperactivation and enlargement of the lymphoid compartment was also closely involved in the development of splenomegaly in portal hypertensive rat [4, 6, 7]. Thus, congestive-hyperplastic model is a better interpretation for splenomegaly in PHT rather than simply congestive.

The mammalian target of rapamycin (mTOR) is a ubiquitously expressed serine/threonine kinase that serves as a central regulator of cell metabolism, growth, proliferation and survival [8, 9]. Discoveries that have been made over the last decade show that mTOR signaling pathway plays a pivotal role in immunological processes, angiogenesis [10, 11] and fibrogenesis [12–15]. Impressively, Mejias and Fernandez recently confirmed that mTOR blockade by rapamycin led to a dramatic regression of splenomegaly and a significant decrease of mesenteric pathological angiogenesis in a non-cirrhotic PHT model [7, 16], indicating the close relevance of mTOR signaling pathway in the pathophysiology of splenomegaly with chronic PHT. However, PHT is the most frequent and important complication that develops in patients with liver cirrhosis [17], therefore, previous findings demonstrated in a non-cirrhotic PHT model might not fully reveal the actual pathogenetic mechanisms of splenomegaly in the context of liver cirrhotic patients and rats with typical cirrhotic PHT [7].

In view of the above questions, we characterized the pathogenetic mechanisms of splenomegaly in PHT patients and two different experimental models of PHT: rats with intrahepatic PHT induced by common bile duct ligation (BDL), and rats with prehepatic PHT induced by partial portal vein ligation (PPVL). This study also systematically determined the role of mTOR signaling pathway during the development of splenomegaly and identified the possible target for therapeutic intervention.

## Materials and Methods

### Ethics statement

All research protocols regarding human samples were approved by the Clinical Review Board and Ethics Committee of Ruijin Hospital. All participating patients were thoroughly informed about the studies and provided written informed consent. All animal care and experimental procedures complied with the guidelines for the Care and Use of Laboratory Animals, formulated by the Ministry of Science and Technology of the People’s Republic of China, and were approved by the Ethical Committee on Animal Experiments at Ruijin Hospital (protocol approval number SYXK 2011–0113).

### Methods

Antibodies against 70-kDa ribosomal protein S6 kinase (p70S6K), ribosomal protein S6 (S6), eukaryotic initiation factor 4E-binding protein 1 (4E-BP1) and their phosphorylated forms, as well as glyceraldehyde 3-phosphate dehydrogenase (GAPDH) and peroxidase-conjugated secondary antibodies, were purchased from Cell Signaling Technology (USA). Antibody against α-smooth muscle actin (α-SMA) was purchased from Doka (Denmark). Antibody against interleukin 1-beta (IL1-β), tumor necrosis factor alpha (TNF-α), vascular endothelial growth factor (VEGF), nuclear factor kappa-light-chain-enhancer of activated B cells (NF-κB) and Ki67 were bought from Santa Cruz Biotechnology (USA). Rapamycin was a kind gift from Pfizer Inc (USA).

### Patients

PHT spleen samples were obtained from liver cirrhotic patients who underwent splenectomy in Ruijin Hospital from Jul. 1, 2014 to Dec. 31, 2014 (n = 12), which were defined as PHT group. Meanwhile, control spleen samples were obtained from patients with traumatic spleen rupture who underwent unavoidable spleen removal (n = 3) and patients with distal pancreatic neoplasms who underwent distal pancreatectomy plus splenectomy (n = 2), which were defined as NON-PHT group. Characteristic of patients and spleens are summarized in Table 1. Spleen sizes were measured and spleen samples were fixed in formalin for histological and immunohistochemical analysis and stored at -80°C for protein analysis.

**Table 1:** Characteristic of patients and spleens.

	Liver cirrhosis (n = 12)	Traumatic spleen rupture (n = 3)	Distal pancreatic neoplasms (n = 2)
Pathological diagnosis	Splenomegaly	Normal spleen	Normal spleen
Male/Female	7 / 5	2 / 1	2 / 0
Mean age, year (SD)	56 (7.4)	52 (0)	60 (4.9)
Liver disease etiology (n)	HBV (8); HCV (3); Schistosomiasis (1)	None	None
Mean spleen sizes (length×width×height cm3)	21.8×14.5×6.3	12.3×7.8×2.9	11.2×6.4×3.1

### Animals and rapamycin treatment schedule

Male Sprague–Dawley rats, weighing 230–280 g, were purchased from Shanghai Slaccas Experiment Animal Corporation (Shanghai, China). PHT was induced by BDL or PPVL in rats, while rats underwent sham operation (SHAM) served as control group. Briefly, after anesthesia (100 mg/kg ketamine, 5 mg/kg xylazine, intramuscularly) and median laparotomy, the common bile duct was isolated and resected between a proximal and distal ligature for BDL. For PPVL, the portal vein was isolated and a constricting ligature was placed over a blunt-tipped 20G needle. In control animals (SHAM) the bile duct or the portal vein were similarly manipulated but no resection or ligation were made [18]. No antibiotics were used in our experiments. Rats were housed in a standard animal laboratory with free activity and access to water and chow. They were kept under constant environmental conditions with a 12-hour light-dark cycle. The rats were fasted for 12 hours before surgery.

In the first experimental protocol, rats were sacrificed on days 1, 3, 7, 14, 21 after BDL or PPVL (n = 4, in each group) and also SHAM (n = 4) in order to study splenomegaly development kinetics and mTOR signaling expression pattern in rat spleens. In the second experimental protocol, rapamycin (2 mg kg^-1^day^-1^) was administered to rats (BDL-RAPA: n = 6; PPVL-RAPA: n = 7; SHAM-RAPA: n = 6) by intraperitoneal injections for a 2-week period, starting one week after operation when PHT was fully established. Vehicle (5% dimethyl sulfoxide solution) was injected to rats (BDL-VEH: n = 7; PPVL-VEH: n = 7; SHAM-VEH: n = 7) intraperitoneally in the same dose and time schedule of rapamycin, which served as control study.

### Measurement of portal pressure

Under anesthesia (100 mg/kg ketamine, 5 mg/kg xylazine, intramuscularly), a 24-g cannula needle was introduced into rat’s portal vein and connected to highly sensitive pressure transducer to measure portal pressure (mm Hg), which was recorded by a multichannel computer-based recorder (Power Lab, AD Instruments, Australia). Pressure measurement lasted for 1 min, and the average value was regarded as the portal pressure. Rats were then sacrificed by cervical dislocation for tissue harvest.

### Western blot analysis

Proteins (60–120μg) were separated by SDS–PAGE and subsequently transferred to polyvinylidenefluoride membranes. Membranes were blocked with 5% non-fat dry milk in incubation buffer and incubated with primary antibodies. Bound antibody was detected with peroxidase-linked secondary antibody and a chemiluminescence detection system. Protein expression was normalized to GAPDH expression. Quantification of protein signals was performed using ImageJ software (NIH, Bethesda, MD).

### Histological and Immunohistochemical analysis

Spleen sections were prepared and stained with hematoxylinand eosin (H&E) and Masson trichrome stain using standard methods. For immunohistochemistry, spleen sections were blocked and incubated with primary antibodies and developed with biotinylated secondary antibodies and then incubated with streptavidin-HRP. The sections were then stained with a solution of 3, 3-diaminobenzidine tetrahydrochloride and counter stained with haematoxylin. Sections were visualized by light microscopy and images were acquired. The positive area was quantified using the ImageJ software (NIH, Bethesda, MD). Morphometric results were expressed as percentage of specific positive area in relation to the total counted area and given as means ± SD.

### Statistical analysis

Comparison between multiple groups was performed with the one-way ANOVA Kruskal-Wallis test, and pairwise comparison was performed with Mann Whitney U test. All of the statistical analysis used the Graph Pad statistics software (Graph Pad Software Inc, USA). A *p* value of less than 0.05 was considered significant.

## Results

### Pathophysiology characteristic and mTOR signaling expression profile in spleen of portal hypertensive patient

There are 12 patients in PHT group and 5 patients in NON-PHT group. Characteristic of patients and spleens are summarized in Table 1. The average spleen size was 21.8×14.5×6.3 cm^3^ in PHT group, which was significantly greater than that of NON-PHT group (11.8×7.1×3.0 cm^3^; *p*<0.01).

**Fig 1:**
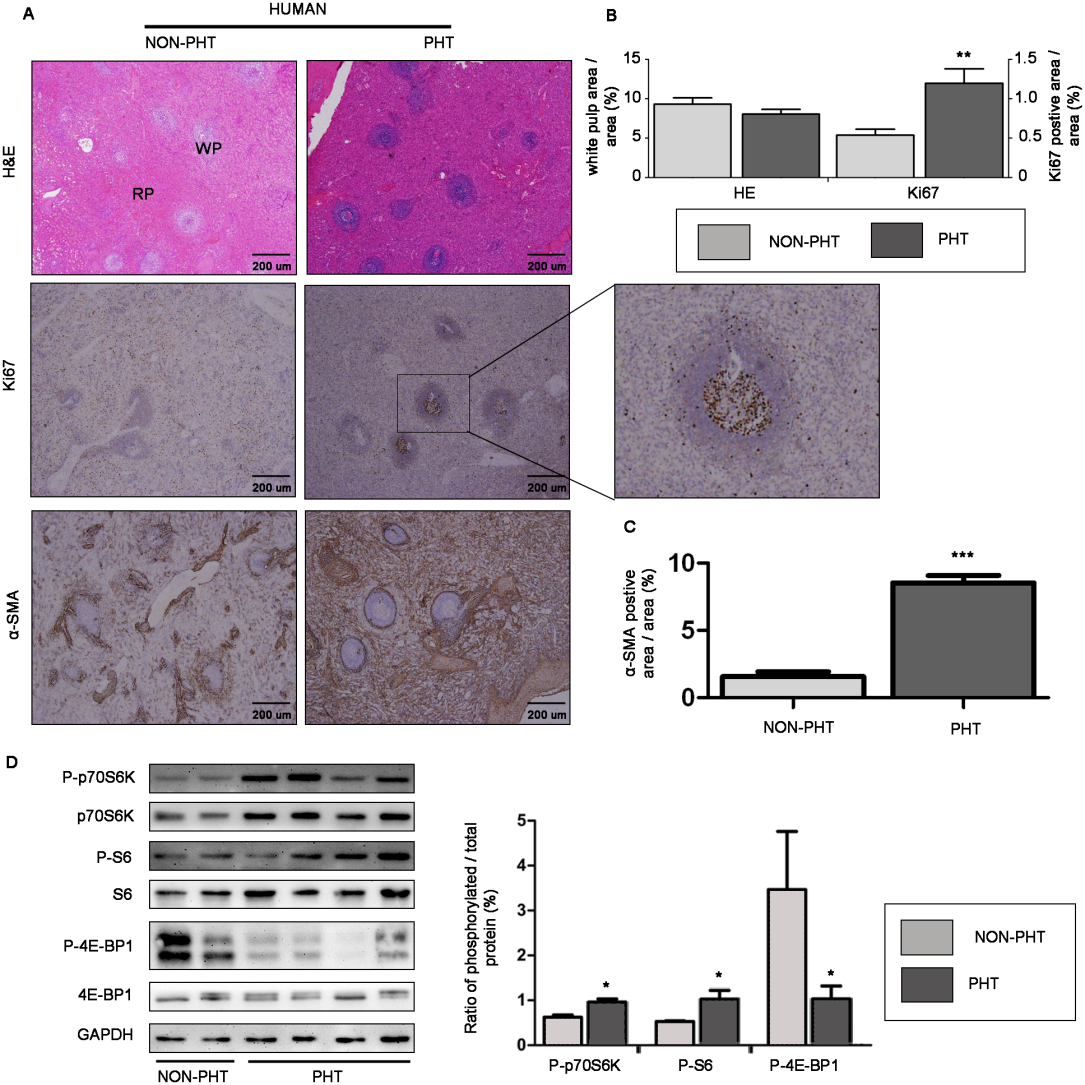
Pathophysiology characteristic and mTOR signaling expression profile in spleens of portal hypertensive patients. (A) Representative images of spleen tissues stained with H&E showing white pulp (WP) and red pulp (RP), and immunostained for Ki67 or α-SMA (original magnification ×40). The image in the middle right is a magnified view of the region in the frame. (B) and (C) Quantitative analysis of white pulp occupied area and Ki67- and α-SMA-positive area. (D) Representative images of western blot for mTOR down-stream effectors and quantification of protein expression relative to their total protein. *p<0.05 versus NON-PHT group, **p<0.01 versus NON-PHT group, ***p<0.001 versus NON-PHT group.

We first studied the pathophysiology of splenomegaly in patients with PHT. In H&E stained sections, the white pulp area, representing the lymphoid tissue of spleen, tended to be smaller in the spleens of PHT group than that of NON-PHT group, albeit without significant difference (*p*>0.05; Fig 1A and 1B). However, in immunostained sections, we found an interesting phenomenon that Ki67-positive area was 2.3 folds greater in the spleens of PHT group than that of NON-PHT group (*p*<0.01; Fig 1A and 1B). Additionally, the Ki67-positive cells localization predominantly correlated with the white pulp region (Fig 1A), suggesting proliferation of splenic lymphocytes [19]. We also detected a robust increase in the expression of perivascular cell marker α-SMA in splenic red pulp of PHT group when compared with NON-PHT group (*p*<0.001; Fig 1A–1C), which meant an increased capillary density in splenomegaly [7].

To investigate mTOR signaling expression profile in splenomegaly, we assessed mTOR down-stream effectors in spleen tissues from patients with PHT by immunoblotting. We detected a significant increased protein expression of P-p70S6K and P-S6 relative to their total proteins in spleens of PHT group compared with NON-PHT group (*p*<0.05; Fig 1D). However, relative P-4E-BP1 protein expression was not augmented in spleens of PHT group, but rather much lower than that of NON-PHT group (Fig 1D). These results suggested mTOR signaling pathway was overactivated, at least in p70S6K/S6 down-stream [20], in spleens of portal hypertensive patients.

### Splenomegaly development kinetics and mTOR signaling expression pattern in spleen of portal hypertensive rat

**Fig 2:**
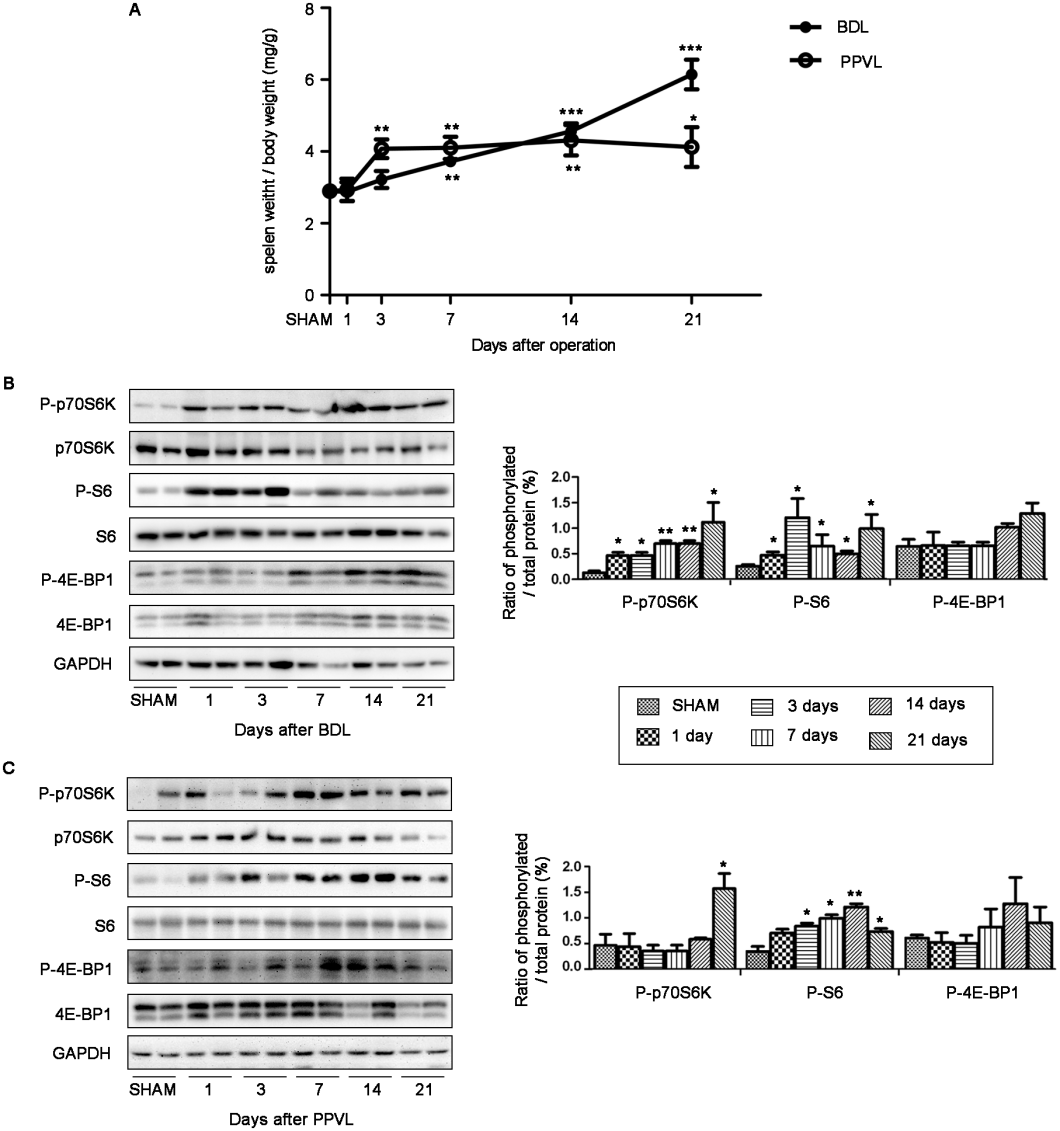
Splenomegaly development kinetics and mTOR signaling expression pattern in spleens of portal hypertensive rats. (A) Relative spleen weight of rats on days 1, 3, 7, 14 and 21 after BDL or PPVL (n = 4 in each group). (B) and (C) Representative images of western blot and quantification of protein expression relative to their total protein for mTOR down-stream effectors in spleens of BDL and PPVL rats. *p<0.05 versus SHAM, **p<0.01 versus SHAM, ***p<0.001 versus SHAM.

To get a better understanding of splenomegaly development kinetics in portal hypertensive rats, we continuously observed pathologic progress at various stages (days 1, 3, 7, 14 and 21 after BDL or PPVL). Interestingly, we found that spleen size increased time-dependently in both rat models of PHT. The ratio of splenic weight to body weight, a measure of spleen size, kept increasing progressively and reached significantly greater on day 7 after BDL when compared with SHAM rats (*p*<0.05; Fig 2A), and kept elevating throughout the next 2 weeks. Splenomegaly peaked maximal level on day 3 and remained high throughout the next 2 weeks in PPVL rats (Fig 2A).

We also studied mTOR signaling expression pattern in spleens of portal hypertensive rats. By immunoblotting, we detected relative P-p70S6K protein expression elevated gradually from day 1 and peaked on day 21 in BDL rat spleens, while remained steady until day 14 and peaked on day 21 in PPVL rat spleens (Fig 2B and 2C). Relative P-S6 protein expression in spleens reached maximal level on day 3 and day 14 in BDL and PPVL rat spleens, respectively, then subsequently decreased (Fig 2B and 2C). Relative P-4E-BP1 protein expression remained unchanged at the first few days then slightly elevated from day 14 in both models, albeit without significant difference from SHAM rats (*p*>0.05; Fig 2B and 2C). These results indicated splenomegaly developed progressively in a time-dependent manner, which might be relevant with mTOR signaling pathway progressive activation at various stages of disease progress.

### mTOR blockade by rapamycin in spleen of portal hypertensive rat

**Fig 3:**
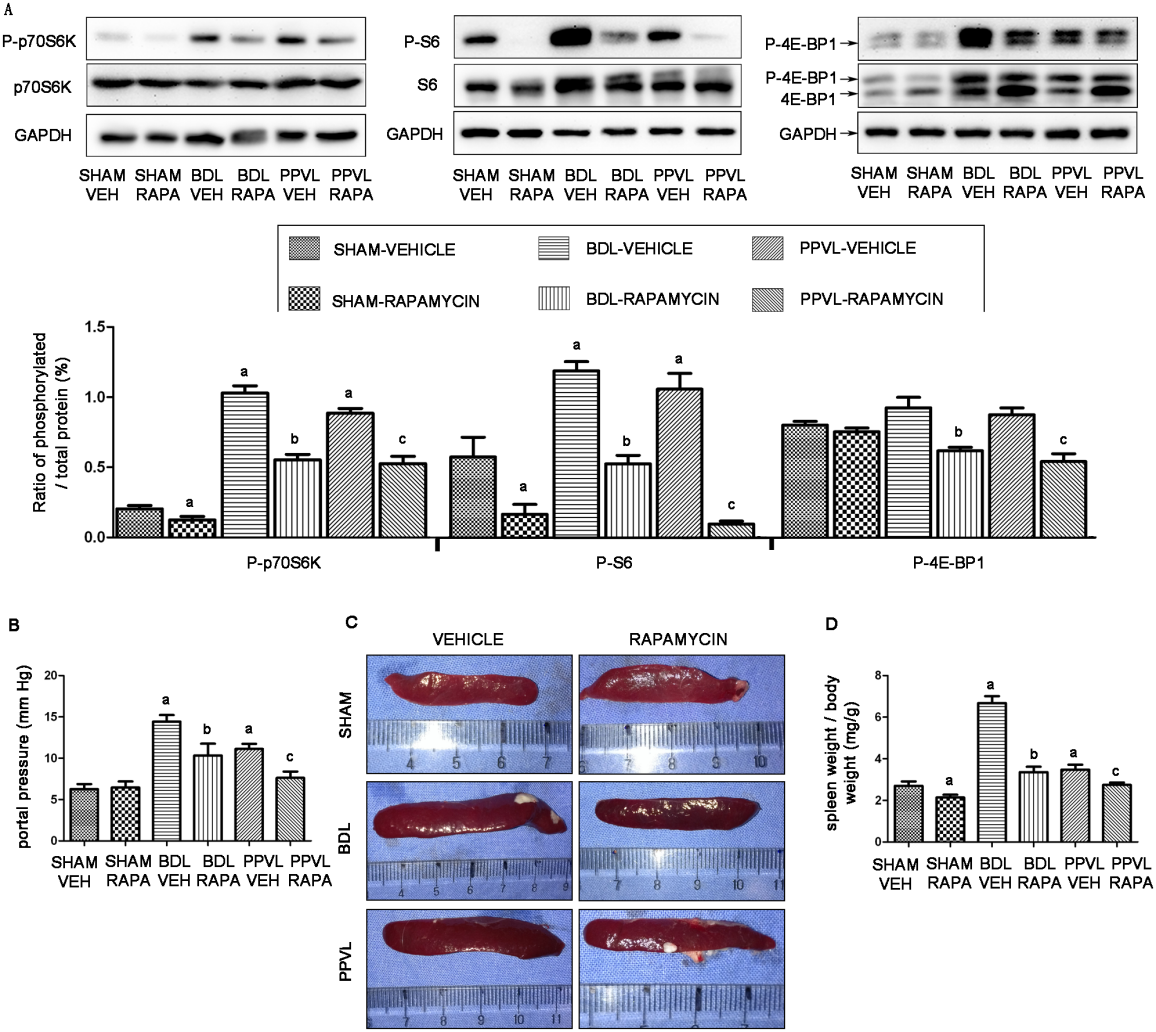
Effects of rapamycin on splenomegaly of portal hypertensive rats. (A) Representative images of western blot and quantification of protein expression relative to their total protein for mTOR down-stream effectors in rat spleens. (B) Portal pressure. (C) Representative spleen images. (D) Quantitative analysis of relative spleen weight. *: *p*<0.05 versus SHAM-VEH, **: *p*<0.05 versus BDL-VEH, ***: *p*<0.05 versus PPVL-VEH.

We chose 3 weeks as our study period and further determined whether mTOR signaling pathway would be altered by 2-week rapamycin treatment, starting one week after BDL or PPVL. Indeed, using the western blot technique, we found that relative P-p70S6K and P-S6 protein expression elevated in vehicle-treated PHT rat spleens were strongly suppressed by rapamycin in BDL-RAPA and PPVL-RAPA rat spleens (*p*<0.05; Fig 3A). Despite relative P-4E-BP1 protein expression did not significantly differed between vehicle-treated PHT rats and SHAM-VEH animals, it was remarkably suppressed by rapamycin in BDL-RAPA and PPVL-RAPA rat spleens (*p*<0.05; Fig 3A). These results suggested that mTOR signaling pathway could be blocked by rapamycin in spleens of portal hypertensive rats.

### mTOR blockade by rapamycin ameliorates portal pressure and splenomegaly

We further gauged the impact of mTOR blockade by rapamycin on splenomegaly. Three weeks after operation, PHT was successfully established along with enlargement of spleen, as indicated by a significant increase in portal pressure and spleen size in BDL-VEH and PPVL-VEH rats, compared to SHAM-VEH rats (*p*<0.05; Fig 3B and 3C). Impressively, two-week treatment of rapamycin induced a 28.3% and 31.4% decrease in portal pressure in BDL-RAPA and PPVL-RAPA rats, respectively, compared to their corresponding vehicle-treated animals (*p*<0.05; Fig 3B). Meanwhile, splenomegaly was profoundly ameliorated by rapamycin in both PHT models, which translated into a 49% and 21% reduction in the ratio of spleen weight to body weight in BDL-RAPA and PPVL-RAPA rats, respectively (*p*<0.05; Fig 3C and 3D).

### mTOR blockade by rapamycin attenuates the enlarged splenic lymphoid tissue

**Fig 4:**
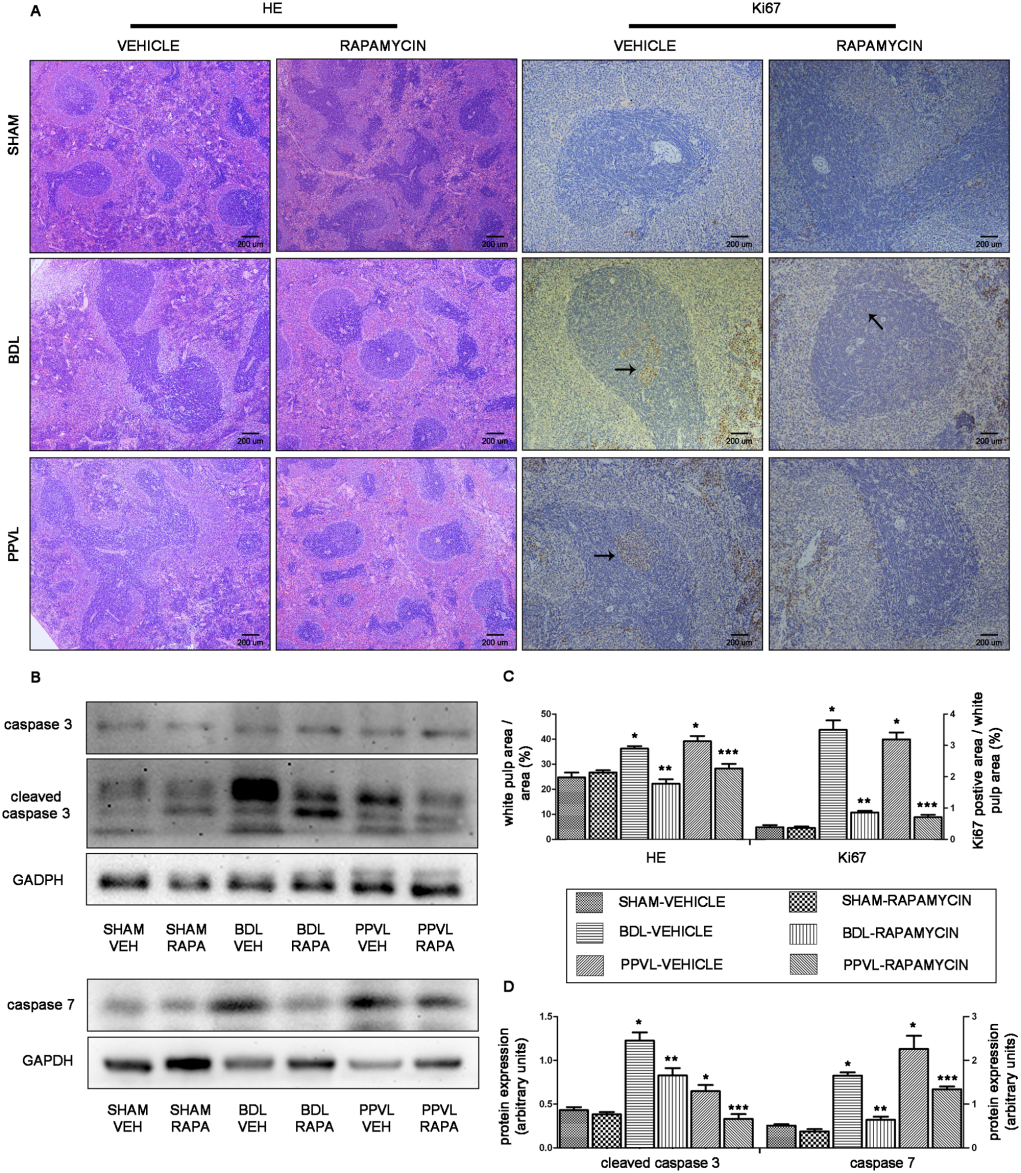
Effects of rapamycin on the splenic lymphoid tissue in portal hypertensive rats. (A) Representative histological images of spleen tissues stained with H&E (original magnification ×40) and immunostained for Ki67 (original magnification ×100). Arrowheads point to Ki67-positive cells. (B) Representative images of western blot for caspase 3 and caspase 7. (C) Quantitative analysis of white pulp occupied area and Ki67-positive cells area to white pulp area. (D) Quantification of cleaved caspase 3 and caspase 7 protein expression relative to GAPDH. *: *p*<0.05 versus SHAM-VEH, **: *p*<0.05 versus BDL-VEH, ***: *p*<0.05 versus PPVL-VEH.

Spleen is an important immune organ, of which the lymphoid tissue, namely white pulp, executes the major immunologic function [21]. We explored the pathophysiology of splenic lymphoid tissue in rats with PHT, and determined the effects of rapamycin on splenic lymphoid tissue. Spleen sections (H&E) from BDL-VEH and PPVL-VEH rats demonstrated an evident enlargement of the splenic lymphoid tissue (Fig 4A). Morphometric quantitative analysis showed a significant larger white pulp area in BDL-VEH and PPVL-VEH rats than that in SHAM-VEH animals (*p*<0.05; Fig 4C). Abundant Ki67-positive cells were also found in the enlarged splenic lymphoid tissue, suggesting splenic lymphocytes were undergoing proliferation (Fig 4A and 4C).

After 2-week intervention, rapamycin markedly attenuated the enlargement of splenic lymphoid tissue, as indicated by a 34% decrease of white pulp area in BDL-RAPA rats and a 35% decrease in PPVL-RAPA rats compared with their corresponding vehicle-treated animals (*p*<0.05; Fig 4A and 4C). Consistently, Ki67-positive cells in white pulp region were markedly reduced in rapamycin-treated PHT rats (Fig 4A and 4C). These results indicated anti-proliferation effect of rapamycin on splenic lymphocytes.

We also investigated whether rapamycin would trigger apoptosis and further conduce to white pulp area attenuation. Surprisingly, we found two executioner proteins of apoptosis, cleaved caspase 3 and caspase 7 [22], were apparently upregulated in spleens of vehicle-treated PHT rats (Fig 4B and 4D), which hinted lymphocytes underwent apoptosis during splenomegaly developed. After 2-week treatment of rapamycin, cleaved caspase 3 and caspase 7 expression were significantly downregulated in spleens of BDL-RAPA or PPVL-RAPA rats (*p*<0.05; Fig 4B and 4D), suggesting that this treatment inhibited lymphocytes apoptosis rather than triggered apoptosis.

### mTOR blockade by rapamycin suppresses pathological angiogenesis in splenic red pulp

**Fig 5:**
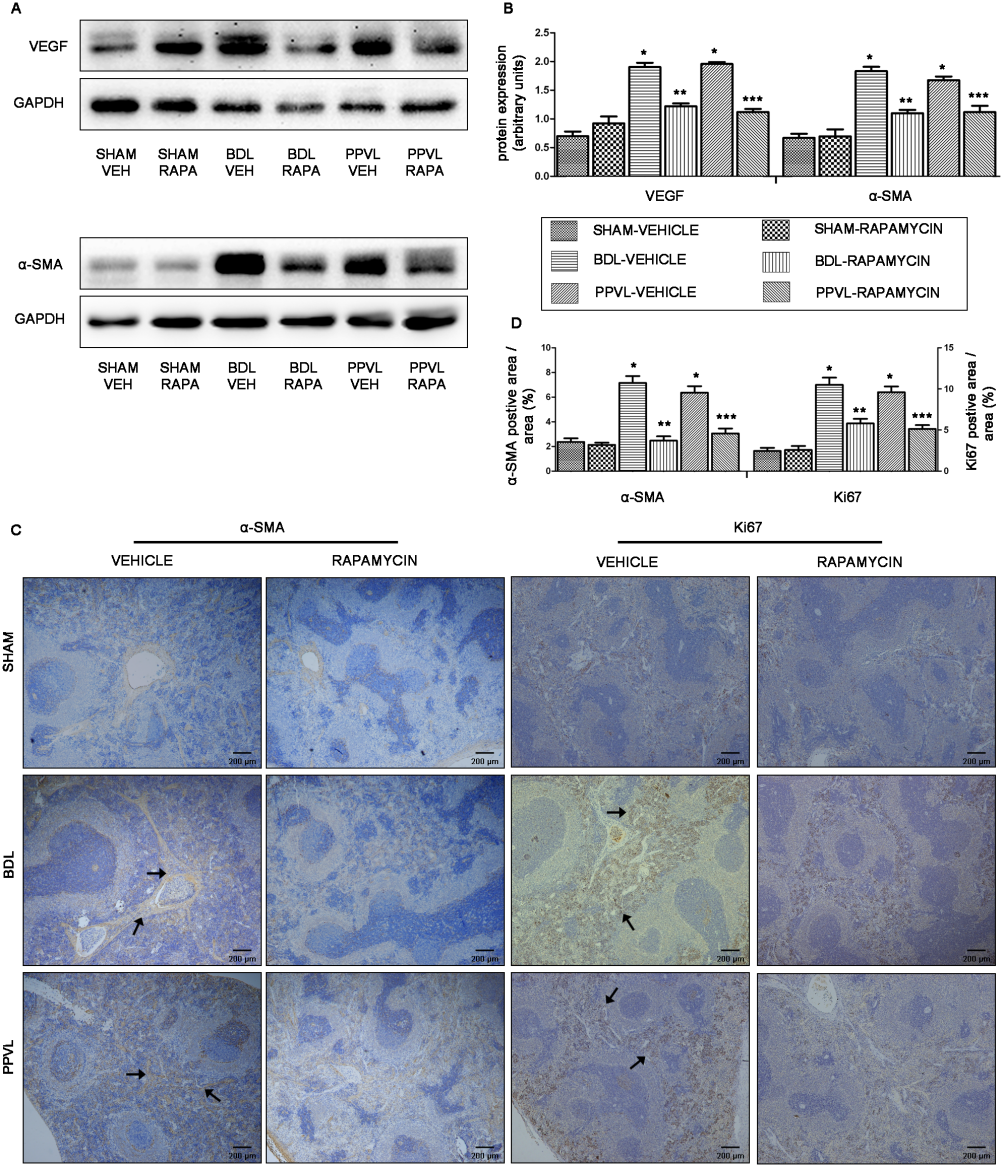
Effects of rapamycin on angiogenesis of splenic red pulp in portal hypertensive rats. (A) Representative images of western blot for VEGF and α-SMA. (B) Quantification of VEGF and α-SMA protein expression relative to GAPDH. (C) Representative histological images of spleen tissues immunostained for α-SMA and Ki67 (original magnification ×40). Arrowheads point to α-SMA- and Ki67-positive cells. (D) Quantitative analysis of α-SMA and Ki67-positive cells area. *: *p*<0.05 versus SHAM-VEH, **: *p*<0.05 versus BDL-VEH, ***: *p*<0.05 versus PPVL-VEH.

As angiogenesis is essential in development and maintenance of PHT [18, 23, 24], we tried to inspect the relevance of angiogenesis in the pathophysiology of splenomegaly in portal hypertensive rats. By immunoblotting, we found a robust increase in the expression of proangiogenic factor VEGF and perivascular cell marker α-SMA [7, 25] in spleens of BDL-VEH and PPVL-VEH rats, compared to SHAM-VEH animals (*p*<0.05; Fig 5A and 5B). By immunostaining, the splenic red pulp of BDL-VEH and PPVL-VEH rats exhibited a significant increase in α-SMA-positive area (*p*<0.05; Fig 5C and 5D), which indicated an increased neovascularization in splenic red pulp. Additionally, the Ki67-positive cells were pronouncedly upregulated in the splenic red pulp of BDL-VEH and PPVL-VEH rats, where it was consistent with sinusoidal endothelial cells (*p*<0.05; Fig 5C and 5D).

After 2-week treatment of rapamycin, VEGF and α-SMA were markedly suppressed in spleens of BDL-RAPA and PPVL-RAPA rats, compared to vehicle-treated PHT animals (*p*<0.05; Fig 5A and 5B). The α-SMA-positive area and Ki67-positive cells, exhibited in splenic red pulp, were also strongly decreased in rapamycin-treated PHT rats (Fig 5C and 5D). Taken together, these data suggested that pathological angiogenesis was involved in the pathophysiology of splenomegaly in portal hypertensive rats, which could be suppressed by rapamycin intervention.

### mTOR blockade by rapamycin improves fibrosis and inflammation in splenic parenchyma

**Fig 6:**
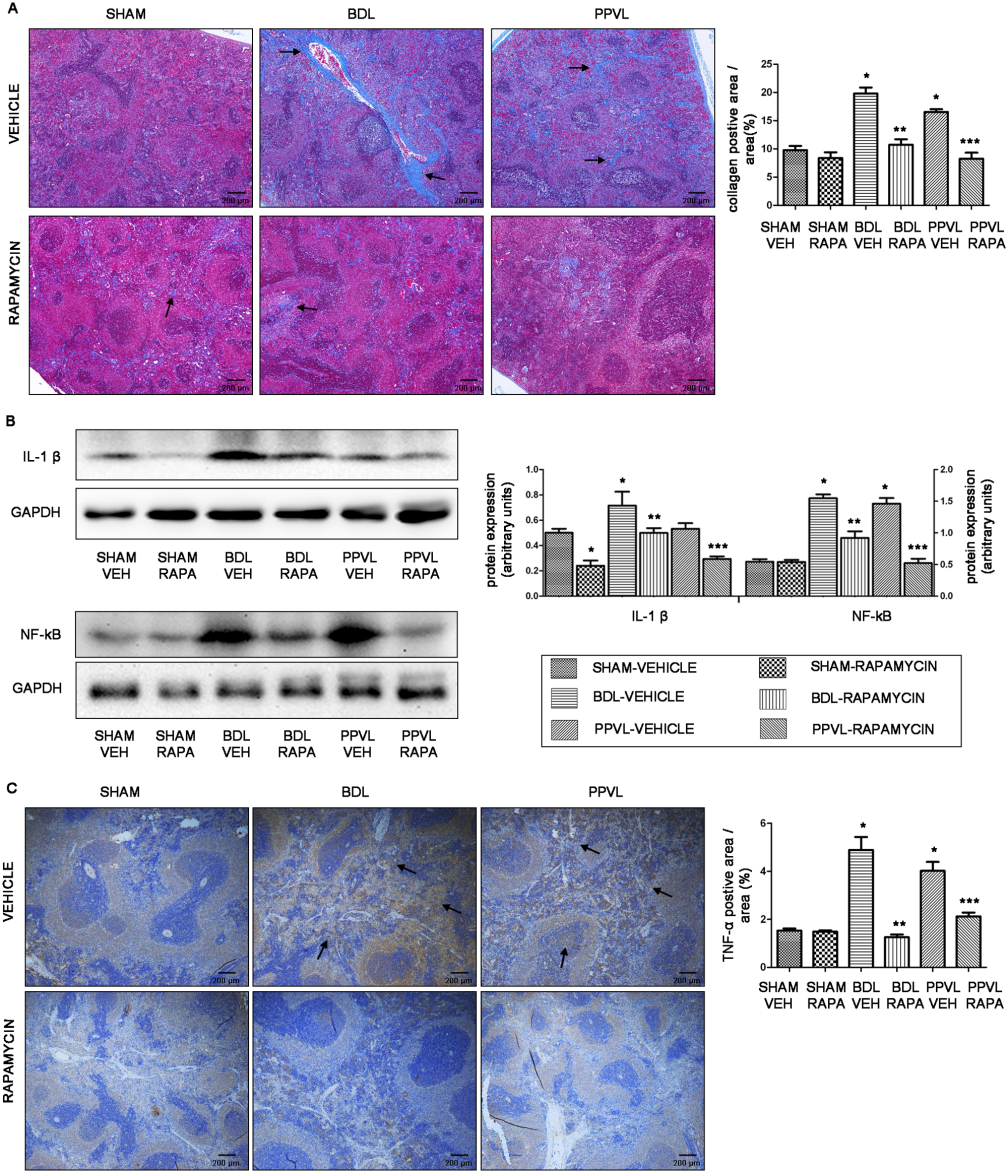
Effects of rapamycin on fibrosis and inflammation in splenic parenchyma in portal hypertensive rats. (A) Representative Masson trichrome staining images (original magnification ×40) and quantitative analysis of fibrillar collegan-stained area. Arrowheads point to fibrillar collagen. (B) Representative images of western blot and quantification of protein expression relative to GAPDH for IL-1β and NF-κB. (C) Representative images of spleen tissues immunostained for TNF-α (original magnification ×40) and quantitative analysis of TNF-α-positive cells area. Arrowheads point to TNF-α-positive cells. *: *p*<0.05 versus SHAM-VEH, **: *p*<0.05 versus BDL-VEH, ***: *p*<0.05 versus PPVL-VEH.

The increased numbers of fibrillar collagen have been found extending to the entire parenchyma in the enlarged spleen of liver cirrhosis previously [26, 27]. Accordantly, as evidenced by Masson trichrome staining, we also found that a 112% and 69% increase in fibrillar collagen presented in splenic parenchyma of BDL-VEH and PPVL-VEH rats, respectively, when compared with SHAM-VEH animals (*p*<0.05; Fig 6A). As expected, rapamycin substantially reduced the amount of fibrillar collagen in spleens of rapamycin-treated PHT rats (Fig 6A).

Since liver cirrhosis is a complex disease, in which fibrosis, angiogenesis and inflammation are closely integrated [28], we assumed that inflammation also exists in the enlarged spleens of portal hypertensive rats. As expected, IL-1β and NF-κB, two proinflammatory cytokines thought to be involved in many acute and chronic diseases [29, 30], were upregulated in spleens of vehicle-treated PHT rats, though IL-1β was not significantly different between PPVL-VEH and SHAM-VEH rats (*p*>0.05; Fig 6B). In accordance with western blot, immunohistochemical findings also revealed an increased expression of TNF-α in spleens of vehicle-treated PHT rats (Fig 6C). Notably, IL-1β and NF-κB were significantly downregulated in spleens of rapamycin-treated PHT rats as compared with vehicle-treated animals (*p*<0.05; Fig 6B). TNF-α-positive cells was barely found in spleen from rapamycin-treated animals, as quantitative analysis indicated by a 74% and 47% decrease in BDL-RAPA and PPVL-RAPA rats, respectively (*p*<0.05; Fig 6C).

## Discussion

It has long been considered that enlargement of spleen is mainly due to passive splenic congestion [1, 4]. Our present study strongly supplemented the concept that splenomegaly was also caused by increased angiogenesis, fibrogenesis, inflammation and splenic lymphoid tissue proliferation. We also found that splenomegaly developed progressively in a time-dependent manner in rats of both intrahepatic and prehepatic portal hypertension models, which was closely in relevance with progressive activation of mTOR signaling pathway. mTOR blockade by rapamycin could profoundly ameliorate splenomegaly by limiting lymphocytes proliferation, angiogenesis, fibrogenesis and inflammation as well as decreasing portal pressure in portal hypertensive rats.

Our previous study and emerging experimental data have already demonstrated that mTOR signaling pathway played a central role in the activation of hepatic stellate cells, moreover, the pathway blockade by rapamycin could reverse liver fibrosis, improve liver function, and lower portal pressure in established cirrhotic animal models [12–15]. Consistently, these reverse effects were also observed in splenomegaly in our experiments, which indicated that mTOR signaling pathway might be systematically activated in portal hypertension syndrome, and rapamycin could be a promising agent in reversing portal hypertension syndrome.

In human spleens, even though the splenic lymphoid tissue region per unit of weight was similar between the two groups, the spleen size was significantly greater in portal hypertensive patients than that in non-portal hypertensive patients. Thus, it was reasonable to assume that an overall increase in the absolute splenic lymphoid tissue might contribute to the enlargement of spleen in patients with PHT. We also found the Ki67-positive cells localization predominantly correlated with the white pulp region, which is a solid evidence of proliferation of splenic lymphoid tissue [19]. In addition, the increased α-SMA-positive area in spleens of PHT patients was a compelling sign of neovascularization in splenic red pulp [25]. Taken together, splenomegaly in patients with PHT could be, in part, the result of overall increase in the absolute splenic lymphoid tissue as a consequence of cellular proliferation and increased neovascularization in the splenic red pulp.

These findings in human spleens were further proved in our animal experiments. Spleens of BDL and PPVL rats exhibited an evident enlargement of the splenic lymphoid tissue along with abundant Ki67-positive proliferating cells, indicating proliferation of splenic lymphocytes [19]. Nevertheless, increased lymphocytes apoptosis in PHT rat spleens discovered in our study was a discrepancy to Mejias’ research that lymphocytes apoptosis did not significantly alter under PHT induction [7]. Moreover, suppressed splenic lymphocytes apoptosis was found in our study upon rapamycin treatment, while augmented apoptosis in Mejias’ research [7]. Recent accumulated researches indicated that liver cirrhosis is a wound-healing response in face with sustained injuries, such as inflammatory factors, oxidative stress and nitrosative stress, by which may induce cellular mitochondrial injury, apoptosis and even necrosis [17, 31]. Furthermore, besides as a consequence of liver injury, apoptosis of parenchymal cells is also viewed as a critical inflammatory stimulus that activates stellate cells [32, 33]. Indeed, apoptosis were significantly triggered in BDL rat livers, as evidenced in the study of Alessandro Arduini [33]. In our previous study, mitochondrial injury and cellular apoptosis, as evidenced by transmission electron microscopy, were also obvious in BDL rat livers. However, treatment with rapamycin markedly modified microenvironment and attenuated mitochondrial injury and cellular apoptosis [15]. From the microenvironment perspective, it is extremely likely that sustained injuries can trigger lymphocytes apoptosis in the progress of splenomegaly with PHT, and lymphocytes apoptosis can be suppressed once the lesion is alleviated by rapamycin.

Our results also demonstrated a significant upregulation of proangiogenic factor VEGF and perivascular cell marker α-SMA in spleens of rats with PHT [19, 25], suggesting increased pathological angiogenesis in the spleens. These findings were further confirmed by the evidence of increased α-SMA-positive area and Ki67-positive cells in splenic red pulp. We also found extensive fibrillar collagen extended to the entire splenic parenchyma in rats with PHT, as evidenced by Masson trichrome staining. Additionally, inflammation was obvious in spleens of the two different models of PHT, as shown by the increase of proinflammatory cytokines IL-1β, NF-κB and TNF-α in splenic parenchyma [29, 30]. It is well acknowledged that angiogenesis, fibrosis and inflammation are closely integrated disturbances in liver cirrhosis, which play important roles in pathophysiology of liver cirrhosis [31, 34]. For instance, many inflammatory mediators may indirectly stimulate other cells producing angiogenic factors to activate angiogenic process [35]. Angiogenesis, in turn, contributes to the recruitment and infiltration of inflammatory cells. The new vessels acting as transporters of nutrients to the site of inflammation can also accentuate the inflammatory response [28, 35, 36]. Therefore, these three disturbances presented in splenomegaly may not only exist in isolation, their complicated interactions would promote the development of splenomegaly.

This improved understanding in pathophysiology of splenomegaly ignited our interest to investigate the underlying pathogenetic mechanisms. We shed light on mTOR signaling pathway for its property on regulating cell growth and proliferation, and pivotal role in immunological processes [8, 9], angiogenesis [10, 11] and fibrogenesis [12–15]. To assess this potential relevance, we performed immunoblots on spleens to detect the protein expression of p70S6K, S6 and 4E-BP1, which are direct down-stream targets of the mTOR kinase and whose phosphorylation states are convenient and widely used measure of the activity of mTOR [20]. We found that P-p70S6K and P-S6 relative to their total protein were elevated in spleens of PHT patients, suggesting mTOR signaling pathway was overactivated, at least in p70S6K/S6 down-stream, in splenomegaly of portal hypertensive patients. Consistently, a progressive activation of mTOR signaling pathway was detected in rats after BDL or PPVL. We also found that the spleen size increased progressively in a time-dependent manner, which was basically in parallel with the tendency of the activation of mTOR signaling pathway. As discussed previously [8–15], it is reasonable to envisage that the activation of mTOR signaling pathway contributed to the enlargement of spleen by promoting processes such as proliferation of lymphocytes, angiogenesis and fibrogenesis.

To further identify the relationship between mTOR signaling pathway and pathophysiology of splenomegaly, we determined the effects of mTOR blockade on pathophysiology of splenomegaly. Rapamycin is a potent and exquisitely specific inhibitor of mTOR that has been used clinically in several diseases [11, 37]. Recent studies have demonstrated that rapamycin is able to prevent mesenteric angiogenesis, and reduce spleen volume in portal hypertensive animal models [16, 38]. In renal transplant recipients, rapamycin has been shown to decrease spleen size without compromising splenic function [39]. Thus, rapamycin may be a safe and effective agent in reducing spleen size. After 2-week treatment of rapamycin in our study, the elevated P-p70S6K and P-S6 protein expression relative to their total proteins in PHT rat spleens, as well as relative P-4E-BP1 protein expression, were strongly suppressed. Consequently, the increased portal pressure and spleen size in PHT rats were profoundly attenuated by rapamycin. Splenomegaly was markedly ameliorated by limiting lymphocytes proliferation, angiogenesis, fibrogenesis and inflammation in rats with PHT. Collectively, these results support the notion that activation of mTOR signaling pathway may be one of the pathogenetic mechanisms leading to splenomegaly, and mTOR blockade by rapamycin can effectively ameliorate splenomegaly.

In conclusion, based on clinical investigation and laboratory exploration, our present study provided important insights into the pathogenetic mechanisms of splenomegaly with PHT. Combining the findings reported here and in our previous publication [15], targeting mTOR signaling could represent a potentially effective therapeutic approach for PHT, ameliorating not only intrahepatic disturbances, but also splenomegaly.

## Supporting Information

S1 Fig H&E staining in rat livers. (TIF)

S2 Fig Immunohistochemistry staining for VEGF in rat spleens. (TIF)

S1 Table Hematological analysis. (DOCX)
