# Supplementary figures and images for: Rapamycin Attenuates Splenomegaly in both Intrahepatic and Prehepatic Portal Hypertensive Rats by Blocking mTOR Signaling Pathway
Source: PLoS One. 2016 Jan 6;11(1):e0141159. doi: 10.1371/journal.pone.0141159 (PMC4703391; doi:10.1371/journal.pone.0141159)

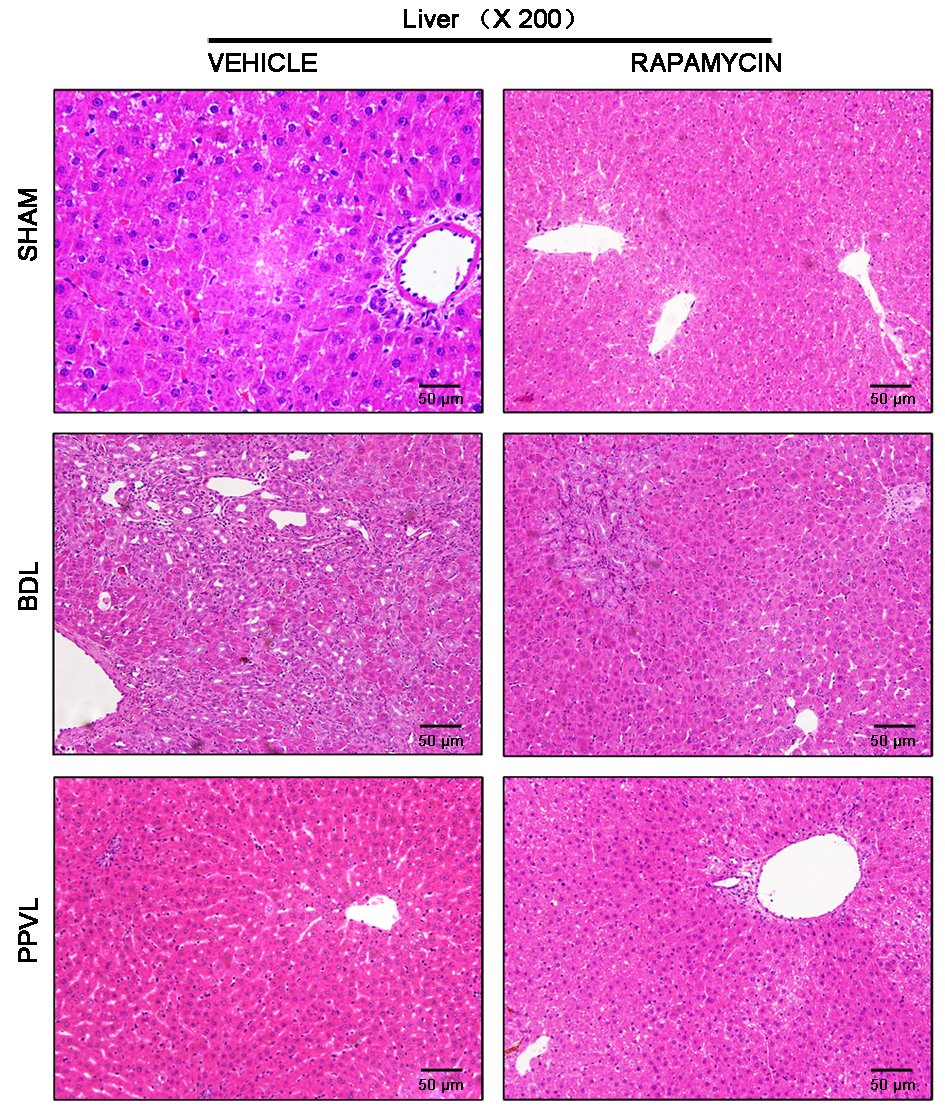

Supplement: S1 Fig — (TIF) [file pone.0141159.s001.tif]

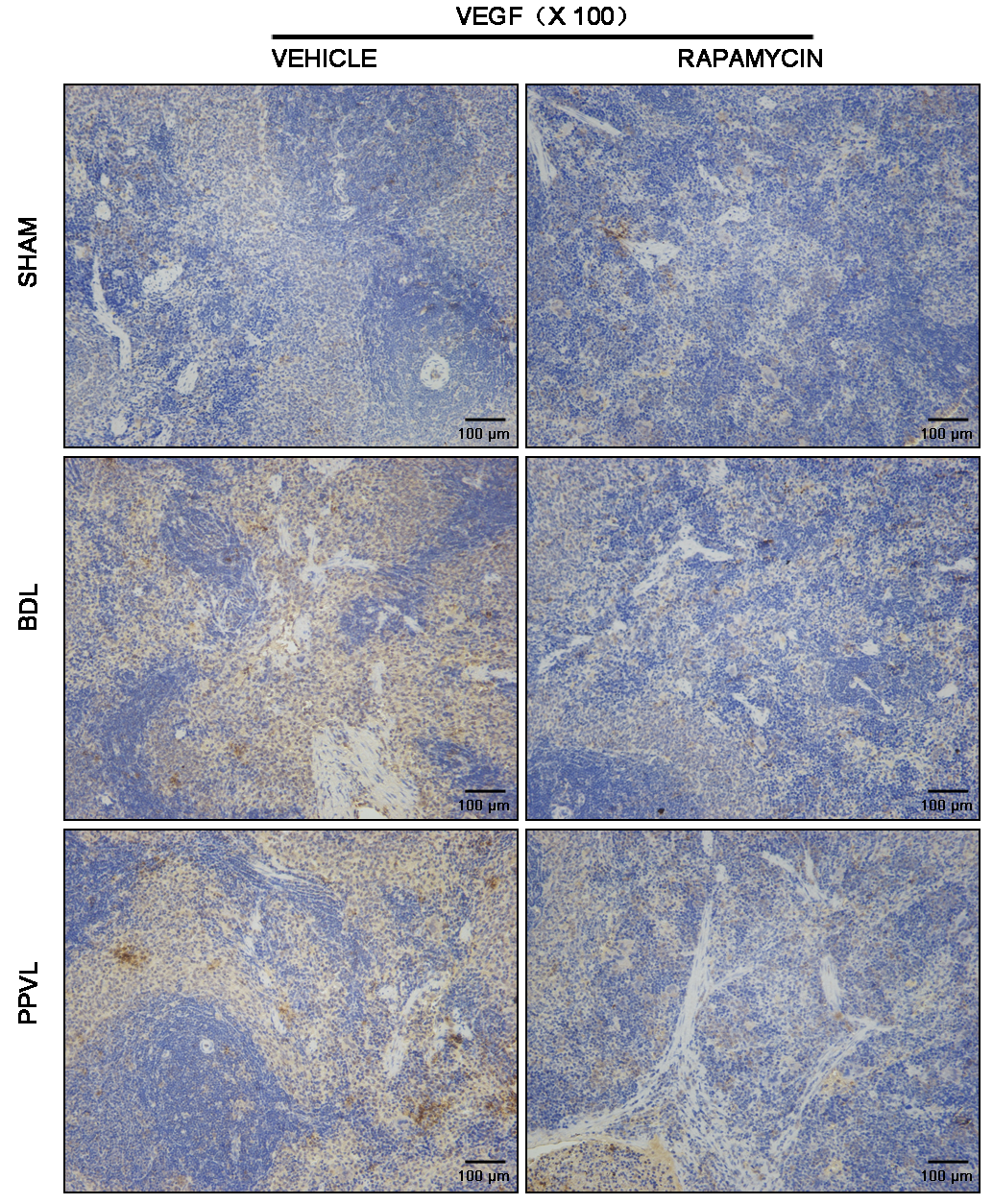

Supplement: S2 Fig — (TIF) [file pone.0141159.s002.tif]
