# Supplementary material for: Rapamycin Attenuates Splenomegaly in both Intrahepatic and Prehepatic Portal Hypertensive Rats by Blocking mTOR Signaling Pathway
Source: PLoS One. 2016 Jan 6;11(1):e0141159. doi: 10.1371/journal.pone.0141159 (PMC4703391; doi:10.1371/journal.pone.0141159)
Supplement: S1 Table — (DOCX) [file pone.0141159.s003.docx]

**S1 Table. Hematological analysis**

|  | SHAM-VEH (n=7) | SHAM-RAPA (n=6) | BDL-VEH (n=7) | BDL-RAPA (n=6) | PPVL-VEH (n=7) | PPVL-RAPA (n=7) |
| --- | --- | --- | --- | --- | --- | --- |
| RBC (×10^12^/L) | 7.60±0.32 | 8.82±0.46* | 6.72±0.64* | 9.68±1.19** | 6.45±0.83* | 8.35±0.63*** |
| HGB (g/L) | 150.0±12.23 | 165.3±24.85* | 125.3±9.07* | 171.0±22.42** | 130.7±11.07* | 159.0±9.90*** |
| WBC (×10^9^/L) | 9.70±0.99 | 8.33±1.56 | 11.32±1.80 | 9.34±0.86 | 9.83±1.79 | 9.45±2.85 |
| PLT (×10^9^/L) | 1370±59.40 | 787±89.41* | 1458±96.3 | 903±101.3** | 1569±132.4 | 608±78.7*** |

**Abbreviations:** RBC, red blood cell; HGB, hemoglobin; WBC, white blood cell; PLT, platelet. Results were expressed as mean ± SD, *: *p*<0.05 versus SHAM-VEH, **: *p*<0.05 versus BDL-VEH, ***: *p*<0.05 versus PPVL-VEH.
